# Supplementary material for: A Bayesian Alternative to Mutual Information for the Hierarchical Clustering of Dependent Random Variables
Source: PLoS One. 2015 Sep 25;10(9):e0137278. doi: 10.1371/journal.pone.0137278 (PMC4583305; doi:10.1371/journal.pone.0137278)
Supplement: S1 File — (PDF) [file pone.0137278.s001.pdf]

Supporting information

A Illustration of the behavior of estimated mutual information

In the case of a D-dimensional variable $\boldsymbol{X}$ with a multivariate normal distribution, the mutual information between two subvectors $\boldsymbol{X}_i$ and $\boldsymbol{X}_j$ is given by:

$$I(\boldsymbol{X}_i, \boldsymbol{X}_j) = \frac{1}{2} \ln \frac{|\boldsymbol{\Sigma}_i|\,|\boldsymbol{\Sigma}_j|}{|\boldsymbol{\Sigma}_{i \cup j}|}, \tag{A1}$$

where $\boldsymbol{\Sigma}_k$, $k \in \{i, j, i \cup j\}$, is the covariance matrix of $\boldsymbol{X}_k$ and $|\cdot|$ is the usual determinant function.

Estimation bias. In this case, mutual information is estimated using its plug-in estimator $\hat{I}$, that is, Equation (A1) where the model covariance matrices have been replaced by their estimators (i.e., the corresponding sample covariance matrices). For large N, this estimator suffers from a systematic bias, as it was shown that [1]

$$\mathrm{E}(\hat{I}) = I(\boldsymbol{X}_i, \boldsymbol{X}_j) + \frac{D_i D_j}{2N} + O\left(\frac{1}{N^2}\right).$$

This bias is additive and is a (quadratic) function of the problem dimensionality. This is consistent with the fact that $2N\, I(\boldsymbol{X}_i, \boldsymbol{X}_j)$ asymptotically follows a chi square distribution with $D_i D_j$ degrees of freedom [2, Chap. 12, §3.6]; [3, §11.3.2].

Extensivity of the measure. Besides the above problem of estimation, mutual information itself is an extensive quantity i.e., it mechanically increases with an increase in the dimensionality of the problem. To better demonstrate this effect, consider a model of homogeneous matrix for $\boldsymbol{\Sigma}$. More specifically, let $\boldsymbol{A}_D(\rho)$ be a D-by-D homogeneous matrix with parameter ρ, i.e., a matrix with 1s on the diagonal and all off-diagonal elements equal to ρ. $\boldsymbol{A}_D(\rho)$ has two eigenvalues: $1 + (D-1)\rho$ with multiplicity 1 (associated with the vector composed only of 1s) and $1 - \rho$ with multiplicity $D - 1$ (associated with the subspace of vectors with a zero mean). This covariance matrix is therefore positive definite for $0 \le \rho < 1$ and its determinant is given by $[1 + (D-1)\rho](1-\rho)^{D-1}$. Assuming that $\boldsymbol{\Sigma} = \boldsymbol{A}_D(\rho)$, mutual information can be expressed as

$$I(\boldsymbol{X}_i, \boldsymbol{X}_j) = \frac{1}{2} \ln \frac{[1 + (D_i - 1)\rho][1 + (D_j - 1)\rho]}{(1-\rho)[1 + (D_i + D_j - 1)\rho]}, \tag{A2}$$

where D_k, $k \in \{i, j, i \cup j\}$, is the dimension of $\boldsymbol{X}_k$. This expression can lead to counter-intuitive results: the mutual information is about 0.31 for $D_i = D_j = 5$ and $\rho = 0.3$, while it is about 0.33 for $D_i = D_j = 7$ and $\rho = 0.25$. More generally, $I(\boldsymbol{X}_i, \boldsymbol{X}_j)$ is an increasing function of D_i and D_j (as can be seen by differentiation). This means that, for the same value of ρ, mutual information will be larger for larger values of D_i and D_j. It also means that mutual information will favor the merging of two variables with smaller marginal correlations if the dimensionality of the variables is large enough, thus systematically favoring larger clusters. To give a feeling of

the amplitude of this behavior, set $D'_k = D_k - 1$ and assume that we have $D'_k \rho \gg 1$. Mutual information can then be approximated by

$$I(\boldsymbol{X}_i, \boldsymbol{X}_j) \approx \frac{1}{2} \ln \frac{D'_i D'_j \rho}{(1-\rho) D'_{i \cup j}} = \frac{1}{2} \ln \frac{\rho}{1-\rho} + \frac{1}{2} \ln \frac{D'_i D'_j}{D'_{i \cup j}}.$$

B Asymptotic form of the log Bayes factor

Assume that $N \to \infty$. Starting from the expression ϕ of Equation (15), we have

$$\phi(N + \nu_k, \boldsymbol{\Lambda}_k + \boldsymbol{S}_k) = -\frac{N + \nu_k}{2} \ln |\boldsymbol{\Lambda}_k + \boldsymbol{S}_k| + \sum_{d=1}^{D_k} \ln \Gamma\left(\frac{N + \nu_k + 1 - d}{2}\right). \quad \text{(B1)}$$

Defining $\widehat{\boldsymbol{S}}_k$ as the standard sample covariance matrix, i.e., $\boldsymbol{S}_k = N \widehat{\boldsymbol{S}}_k$, the first term of the right-hand side can be expanded as

$$\begin{aligned}
\frac{N + \nu_k}{2} \ln |\boldsymbol{\Lambda}_k + \boldsymbol{S}_k| &= \frac{N + \nu_k}{2} \ln |\boldsymbol{\Lambda}_k + N \widehat{\boldsymbol{S}}_k| \\
&= \left(\frac{N}{2} + \frac{\nu_k}{2}\right) \left[D_k \ln N + \ln |\widehat{\boldsymbol{S}}_k| + \ln |\boldsymbol{I} + (N \widehat{\boldsymbol{S}}_k)^{-1} \boldsymbol{\Lambda}_k|\right] \\
&= \frac{D_k N}{2} \ln N + \frac{N}{2} \ln |\widehat{\boldsymbol{S}}_k| + \frac{D_k \nu_k}{2} \ln N + O(1),
\end{aligned}$$

since $|a\boldsymbol{A}| = a^{\dim(\boldsymbol{A})} |\boldsymbol{A}|$ for any positive number a and matrix $\boldsymbol{A}$. In the expression of ϕ in Equation (B1), each term in the sum can be approximated using Stirling approximation [4, p. 257]

$$\ln \Gamma(z) = \left(z - \frac{1}{2}\right) \ln z - z + O(1).$$

Setting $z = (N + \nu_k + 1 - d)/2$ for $l \in \{i, j, i \cup j\}$, we obtain

$$\ln \Gamma\left(\frac{N + \nu_k + 1 - d}{2}\right) = \frac{N + \nu_k - d}{2} \ln N - \frac{N}{2}(1 + \ln 2) + O(1).$$

Summing this expression over $d = 1, \ldots, D_k$ and using the fact that $\sum_{d=1}^{D_k} d = D_k(D_k + 1)/2$ leads us to

$$\begin{aligned}
\sum_{d=1}^{D_k} \ln \Gamma\left(\frac{N + \nu_k + 1 - d}{2}\right) &= D_k \left[\frac{N + \nu_k}{2} \ln N - \frac{N}{2}(1 + \ln 2)\right] \\
&\quad - \frac{D_k(D_k + 1)}{4} \ln N + O(1).
\end{aligned}$$

We then have for ϕ

$$\phi(N + \nu_k, \boldsymbol{\Lambda}_k + \boldsymbol{S}_k) = -\frac{N}{2} \ln |\widehat{\boldsymbol{S}}_k| - \frac{D_k N}{2}(1 + \ln 2) - \frac{D_k(D_k + 1)}{4} \ln N + O(1). \quad \text{(B2)}$$

Since $\phi(\nu_k, \boldsymbol{\Lambda}_k)$ does not depend on N, it is $O(1)$, and the Taylor expansion of $\Delta\phi_k$, as defined in Equation (14), is also given by Equation (B2). Finally, the approximation for $s(\boldsymbol{X}_i, \boldsymbol{X}_j)$ of Equation (11) is given by

$$\begin{aligned} s(\boldsymbol{X}_i, \boldsymbol{X}_j) &= \frac{N}{2} \ln \frac{|\widehat{\boldsymbol{S}}_i|\,|\widehat{\boldsymbol{S}}_j|}{|\widehat{\boldsymbol{S}}_{i\cup j}|} - \frac{1}{2}\left[\frac{D_{i\cup j}(D_{i\cup j}+1)}{2} - \sum_{k\in\{i,j\}} \frac{D_k(D_k+1)}{2}\right] \ln N + O(1) \\ &= N\,\hat{I}(\boldsymbol{X}_i, \boldsymbol{X}_j) - \frac{D_i D_j}{2} \ln N + O(1), \end{aligned} \tag{B3}$$

where we used the fact that $D_{i\cup j} = D_i + D_j$, and where we set

$$\hat{I}(\boldsymbol{X}_i, \boldsymbol{X}_j) = \frac{1}{2} \ln \frac{|\widehat{\boldsymbol{S}}_i|\,|\widehat{\boldsymbol{S}}_j|}{|\widehat{\boldsymbol{S}}_{i\cup j}|}.$$

C Hyperparameter optimization

Given a clustering $\{\boldsymbol{X}_1, \ldots, \boldsymbol{X}_K\}$ of $\boldsymbol{X}$, optimizing the marginal likelihood with respect to a diagonal $\boldsymbol{\Lambda}$ leads to the optimization of

$$\sum_{d=1}^{D} \frac{\nu - D + D_{k_d}}{2} \ln \Lambda_{dd} - \sum_{k=1}^{K} \frac{N + \nu - D + D_k}{2} \ln |\boldsymbol{S}_k + \boldsymbol{\Lambda}_k|,$$

where k_d is the cluster containing X_d. Differentiation with respect to Λ_{dd} leads to

$$\frac{\nu - D + D_{k_d}}{2\Lambda_{dd}} - \frac{N + \nu - D + D_{k_d}}{2} \left[(\boldsymbol{S}_{k_d} + \boldsymbol{\Lambda}_{k_d})^{-1}\right]_{dd} = 0. \tag{C1}$$

To obtain the solution of this equation, we notice that the equation is equivalent to

$$\Lambda_{dd} = \frac{\nu - D + D_{k_d}}{N + \nu - D + D_{k_d}} \left\{\left[(\boldsymbol{S}_{k_d} + \boldsymbol{\Lambda}_{k_d})^{-1}\right]_{dd}\right\}^{-1}.$$

Optimizing at the first level (i.e., with D clusters and $D_{k_d} = 1$) yields

$$\Lambda_{dd} = \frac{\nu - D + 1}{N} S_{dd}.$$

D Real fMRI data: datasets and preprocessing

We used the 'Atlanta' resting-state fMRI database [5]. This resource was made publicly available as part of the 1000-connectome project[1] [6]. The Atlanta sample includes 28 subjects (age ranging from 22 to 57 years, 15 women) with one structural MRI and one fMRI run each (205 volumes, TR = 2 s), acquired on a 3 T scanner. The datasets were preprocessed using the

[1] http://www.nitrc.org/projects/fcon_1000/

neuroimaging analysis kit[2] (NIAK), version 0.6.5c [7]. The parameters of a rigid body motion were first estimated at each time frame of the fMRI dataset (no correction of inter-slice difference in acquisition time was applied). The median volume of the fMRI time series was coregistered with a T_1 individual scan using Minctracc[3] [8], which was itself transformed to the Montreal Neurological Institute (MNI) non-linear template [9] using the CIVET[4] pipeline [10]. The rigid-body transform, fMRI-to-T_1 transform and T_1-to-stereotaxic transform were all combined, and the functional volumes were resampled in the MNI space at a 3 mm isotropic resolution. The "scrubbing" method of [11] was used to remove the volumes with excessive motion (frame displacement greater than 0.5). The following nuisance parameters were regressed out from the time series at each voxel: slow time drifts (basis of discrete cosines with a 0.01 Hz high-pass cut-off), average signals in conservative masks of the white matter and the lateral ventricles as well as the first principal components (95% energy) of the six rigid-body motion parameters and their squares [12]. The fMRI volumes were then spatially smoothed with a 6 mm isotropic Gaussian blurring kernel. Because some of the measures considered in this paper are poorly conditioned when the number of spatial locations is larger than the number of time points (BIC, Infomut and InfomutNorm), the fMRI time series were spatially averaged on each of the areas of the AAL brain template [13]. To further reduce the spatial dimension, only the 81 cortical AAL areas were included in the analysis (excluding the cerebellum, the basal ganglia and the thalamus). The clustering methods were applied to these regional time series. Note that 8 subjects were excluded because there was not enough time points left after scrubbing (a minimum number of 190 volumes was selected as acceptable), and one additional subject had to be excluded because the quality of the T_1-fMRI coregistration was substandard (by visual inspection). A total of 19 subjects was thus actually used in this analysis.

E Model comparison with the concentration matrix

E.1 Hypothesis of dependence

$\boldsymbol{S}_{i\cup j}$ is Wishart distributed with N degrees of freedom and scale matrix $\boldsymbol{\Sigma}_{i\cup j} = \boldsymbol{\Upsilon}_{i\cup j}^{-1}$

$$\mathrm{p}(\boldsymbol{S}_{i\cup j}|\mathcal{M}_D, \boldsymbol{\Upsilon}_{i\cup j}) = \frac{|\boldsymbol{S}_{i\cup j}|^{\frac{N-D_{i\cup j}-1}{2}}}{Z(D_{i\cup j}, N)} |\boldsymbol{\Upsilon}_{i\cup j}|^{\frac{N}{2}} \exp\left[-\frac{1}{2}\mathrm{tr}(\boldsymbol{\Upsilon}_{i\cup j}\boldsymbol{S}_{i\cup j})\right].$$

The prior for $\boldsymbol{\Sigma}_{i\cup j}$ [Equation (4) of the manuscript] directly translates into a prior for $\boldsymbol{\Upsilon}_{i\cup j}$ that is Wishart with $\nu_{i\cup j}$ degrees of freedom and scale matrix $\boldsymbol{\Omega}_{i\cup j} = \boldsymbol{\Lambda}_{i\cup j}^{-1}$

$$\mathrm{p}(\boldsymbol{\Upsilon}_{i\cup j}|\mathcal{M}_D) = \frac{|\boldsymbol{\Omega}_{i\cup j}|^{-\frac{\nu_{i\cup j}}{2}}}{Z(D_{i\cup j}, \nu_{i\cup j})} |\boldsymbol{\Upsilon}_{i\cup j}|^{\frac{\nu_{i\cup j}-D_{i\cup j}-1}{2}} \exp\left[-\frac{1}{2}\mathrm{tr}(\boldsymbol{\Omega}_{i\cup j}^{-1}\boldsymbol{\Upsilon}_{i\cup j})\right].$$

[2] http://wiki.bic.mni.mcgill.ca/index.php/NiakFmriPreprocessing
[3] http://wiki.bic.mni.mcgill.ca/index.php/MinctraccManPage
[4] http://wiki.bic.mni.mcgill.ca/index.php/CIVET

This leads to a marginal likelihood of

$$\begin{aligned} \mathrm{p}(\boldsymbol{S}_{i\cup j}|\mathcal{M}_D) &= \frac{|\boldsymbol{S}_{i\cup j}|^{\frac{N-D_{i\cup j}-1}{2}} |\boldsymbol{\Omega}_{i\cup j}|^{-\frac{\nu_{i\cup j}}{2}}}{Z(D_{i\cup j},N)\, Z(D_{i\cup j},\nu_{i\cup j})} \\ &\quad \times \int |\boldsymbol{\Upsilon}_{i\cup j}|^{\frac{N+\nu_{i\cup j}-D_{i\cup j}-1}{2}} \exp\left\{-\frac{1}{2}\mathrm{tr}\left[\boldsymbol{\Upsilon}_{i\cup j}(\boldsymbol{S}_{i\cup j}+\boldsymbol{\Omega}_{i\cup j}^{-1})\right]\right\} \mathrm{d}\boldsymbol{\Upsilon}_{i\cup j}. \end{aligned}$$

The integrand is proportional to a Wishart distribution with $N+\nu_{i\cup j}$ degrees of freedom and scale matrix $(\boldsymbol{S}_{i\cup j}+\boldsymbol{\Omega}_{i\cup j}^{-1})^{-1}$, leading to

$$\mathrm{p}(\boldsymbol{S}_{i\cup j}|\mathcal{M}_D) = \frac{|\boldsymbol{S}_{i\cup j}|^{\frac{N-D_{i\cup j}-1}{2}}}{Z(D_{i\cup j},N)} \frac{Z(D_{i\cup j},N+\nu_{i\cup j})}{Z(D_{i\cup j},\nu_{i\cup j})} \frac{\left|\left(\boldsymbol{S}_{i\cup j}+\boldsymbol{\Omega}_{i\cup j}^{-1}\right)^{-1}\right|^{\frac{N+\nu_{i\cup j}}{2}}}{|\boldsymbol{\Omega}_{i\cup j}|^{\frac{\nu_{i\cup j}}{2}}}. \tag{E1}$$

E.2 Hypothesis of independence

In the case of independence, the same likelihood holds with the addition that, since $\boldsymbol{\Upsilon}_{i\cup j}$ is block diagonal with blocks $\boldsymbol{\Upsilon}_i$ and $\boldsymbol{\Upsilon}_j$, we have $|\boldsymbol{\Upsilon}_{i\cup j}| = |\boldsymbol{\Upsilon}_i|\,|\boldsymbol{\Upsilon}_j|$ as well as $\mathrm{tr}(\boldsymbol{\Upsilon}_{i\cup j}\boldsymbol{S}_{i\cup j}) = \mathrm{tr}(\boldsymbol{\Upsilon}_i\boldsymbol{S}_i)+\mathrm{tr}(\boldsymbol{\Upsilon}_j\boldsymbol{S}_i)$, leading to a likelihood of

$$\mathrm{p}(\boldsymbol{S}_{i\cup j}|\mathcal{M}_I,\boldsymbol{\Upsilon}_{i\cup j}) = \frac{|\boldsymbol{S}_{i\cup j}|^{\frac{N-D_{i\cup j}-1}{2}}}{Z(D_{i\cup j},N)} \prod_{k\in\{i,j\}} |\boldsymbol{\Upsilon}_k|^{\frac{N}{2}} \exp\left[-\frac{1}{2}\mathrm{tr}(\boldsymbol{\Upsilon}_k\boldsymbol{S}_k)\right].$$

The prior for $\boldsymbol{\Upsilon}_k$ that derives from that of $\boldsymbol{\Upsilon}_{i\cup j}$ is a Wishart distribution with $\nu_{i\cup j}$ degrees of freedom and scale matrix $\boldsymbol{\Omega}_k$ [3, §5.1.2]

$$\mathrm{p}(\boldsymbol{\Upsilon}_k|\mathcal{M}_I) = \frac{|\boldsymbol{\Omega}_k|^{-\frac{\nu_{i\cup j}}{2}}}{Z(D_k,\nu_{i\cup j})} |\boldsymbol{\Upsilon}_k|^{\frac{\nu_{i\cup j}-D_k-1}{2}} \exp\left[-\frac{1}{2}\mathrm{tr}(\boldsymbol{\Omega}_k^{-1}\boldsymbol{\Upsilon}_k)\right].$$

This leads to a marginal likelihood of

$$\begin{aligned} \mathrm{p}(\boldsymbol{S}_{i\cup j}|\mathcal{M}_I) &= \frac{|\boldsymbol{S}_{i\cup j}|^{\frac{N-D_{i\cup j}-1}{2}} |\boldsymbol{\Omega}_k|^{-\frac{\nu_{i\cup j}}{2}}}{Z(D_{i\cup j},N)\, Z(D_i,\nu_{i\cup j})\, Z(D_j,\nu_{i\cup j})} \\ &\quad \times \prod_{k\in\{i,j\}} \int |\boldsymbol{\Upsilon}_k|^{\frac{N+\nu_{i\cup j}-D_k-1}{2}} \exp\left\{-\frac{1}{2}\mathrm{tr}\left[\boldsymbol{\Upsilon}_k(\boldsymbol{S}_k+\boldsymbol{\Omega}_k^{-1})\right]\right\} \mathrm{d}\boldsymbol{\Upsilon}_k. \end{aligned}$$

Each integrand is proportional to a Wishart distribution with $N+\nu_{i\cup j}$ degrees of freedom and scale matrix $(\boldsymbol{S}_k+\boldsymbol{\Omega}_k^{-1})^{-1}$, leading to

$$\mathrm{p}(\boldsymbol{S}_{i\cup j}|\mathcal{M}_I) = \frac{|\boldsymbol{S}_{i\cup j}|^{\frac{N-D_{i\cup j}-1}{2}}}{Z(D_{i\cup j},N)} \prod_{k\in\{i,j\}} \frac{Z(D_k,N+\nu_{i\cup j})}{Z(D_k,\nu_{i\cup j})} \frac{\left|\left(\boldsymbol{S}_k+\boldsymbol{\Omega}_k^{-1}\right)^{-1}\right|^{\frac{N+\nu_{i\cup j}}{2}}}{|\boldsymbol{\Omega}_k|^{\frac{\nu_{i\cup j}}{2}}}. \tag{E2}$$

References

1. Marrelec G, Benali H (2011) Large-sample asymptotic approximations for the sampling and posterior distributions of differential entropy for multivariate normal distributions. Entropy 13: 805–819.

2. Kullback S (1968) Information Theory and Statistics. Dover, Mineola, NY.

3. Press SJ (2005) Applied Multivariate Analysis. Using Bayesian and Frequentist Methods of Inference. Dover, Mineola, 2nd edition.

4. Abramowitz M, Stegun IA, editors (1972) Handbook of Mathematical Functions. Number 55 in Applied Math. National Bureau of Standards.

5. Liu H, Stufflebeam SM, Sepulcre J, Hedden T, Buckner RL (2009) Evidence from intrinsic activity that asymmetry of the human brain is controlled by multiple factors. Proceedings of the National Academy of Sciences of the USA 106: 20499–20503.

6. Biswal BB, Mennes M, Zuo XN, Gohel S, Kelly C, et al. (2010) Toward discovery science of human brain function. Proceedings of the National Academy of Sciences of the USA 107: 4734–4739.

7. Bellec P, Lavoie-Courchesne S, Dickinson P, Lerch JP, Zijdenbos AP, et al. (2012) The pipeline system for Octave and Matlab (PSOM): a lightweight scripting framework and execution engine for scientific workflows. Frontiers in Neuroinformatics 6: 7.

8. Collins DL, Neelin P, Peters TM, Evans AC (1994) Automatic 3D intersubject registration of MR volumetric data in standardized Talairach space. Journal of Computer Assisted Tomography 18: 192–205.

9. Fonov V, Evans AC, Botteron K, Almli CR, McKinstry RC, et al. (2011) Unbiased average age-appropriate atlases for pediatric studies. NeuroImage 54: 313–327.

10. Zijdenbos AP, Forghani R, Evans AC (2002) Automatic ”pipeline” analysis of 3-D MRI data for clinical trials: application to multiple sclerosis. IEEE Transactions on Medical Imaging 21: 1280–1291.

11. Power JD, Barnes KA, Snyder AZ, Schlaggar BL, Ptersen SE (2012) Spurious but systematic correlations in functional connectivity MRI networks arise from subject motion. NeuroImage 59: 2142–2154.

12. Giove F, Gili T, Iacovella V, Macaluso E, Maraviglia B (2009) Images-based suppression of unwanted global signals in resting-state functional connectivity studies. Magnetic Resonance Imaging 27: 1058–1064.

13. Tzourio-Mazoyer N, Landeau B, Papathanassiou D, Crivello F, Etard O, et al. (2002) Automated anatomical labeling of activations in SPM using a macroscopic anatomical parcellation of the MNI MRI single-subject brain. NeuroImage 15: 273–289.
